# Large language models for identifying social determinants of health

**DOI:** 10.1073/pnas.2501506122

**Published:** 2025-03-20

**Authors:** Yu Wang

**Affiliations:** ^a^Fudan Institute for Advanced Study in Social Sciences, Fudan University, Shanghai 200433, China

Large language models, including Bidirectional Encoder Representations from Transformers (BERT) and Generative Pre-trained Transformer (GPT), are competing for dominance in natural language processing (1–3). This ongoing competition extends to real-world applications, including a recent study by Gabriel et al. on identifying social determinants of health (SDoH) (4). The authors proposed a method based on RoBERTa, a variant of BERT, to detect homelessness, food insecurity, and domestic violence with very limited labeled data. Comparing the performance of SDoH classifiers trained on three datasets-GPT-generated notes only, authentic notes from Medical Information Mart for Intensive Care (MIMIC), and a combination of synthetic and MIMIC notes-Gabriel et al. concluded that “supplementing training data with synthetic data may optimize predictive performance for identifying SDoH from the unstructured data present in clinical notes” (4). While we commend their findings and the innovative use of synthetic data, we contend that these conclusions may not readily generalize to other studies for three key reasons.

First, some models in the study (4) show potential training limitations, undermining the reliability of their conclusions. For instance, in the task of detecting food insecurity, a RoBERTa-base model fine-tuned on MIMIC notes yields an Area Under the Receiver Operating Characteristics Curve (ROC-AUC) of 0.13. By comparison, random guessing, such as coin flips, would achieve an ROC-AUC of 0.5. Fine-tuning BERT models requires careful hyperparameter tuning, especially when the training set is small (5). However, this study lacks dedicated validation sets and instead relies solely on test sets, leaving critical hyperparameters, such as the learning rate and the number of training epochs, either at default or fixed values (4).

Second, in cases with very limited data, there is strong reason to believe that few-shot learning with GPT can outperform fine-tuning BERT and its variants. RoBERTa models’ state-of-the-art performance has relied on the availability of sufficiently large training datasets. For example, early experiments (3) typically utilize thousands (6) or tens of thousands of training samples (7), whereas Gabriel et al. employ only 40 to 100 data points for fine-tuning. Additionally, (4) uses the base version of RoBERTa, rather than its larger and more performant counterpart. Considering that GPT models are already approaching the performance of RoBERTa-large models (3), Gabriel et al.’s decision to fine-tune RoBERTa-base models makes it more likely that few-shot prompting with GPT could exceed the performance of these fine-tuned models.

Last, when the dataset contains more data points—such as 300—a fine-tuned RoBERTa-large model may no longer benefit from synthetic data. Previous studies (8) have shown that data augmentation provides limited benefits for RoBERTa-base models when there are 500 or more training samples. Furthermore, research indicates that performance gains diminish as the number of data points increases due to diminishing marginal returns (9). Similarly, while synthetic data can improve performance compared to having no additional data, its contribution is considerably less impactful than that of real data (10). This performance degradation is particularly pronounced when synthetic data are generated through zero-shot learning, as in ref. 4, compared to data generated through few-shot learning (10).
